# Glycation of Human Cortical and Cancellous Bone Captures Differences in the Formation of Maillard Reaction Products between Glucose and Ribose

**DOI:** 10.1371/journal.pone.0117240

**Published:** 2015-02-13

**Authors:** Grażyna E. Sroga, Alankrita Siddula, Deepak Vashishth

**Affiliations:** Department of Biomedical Engineering, Center for Biotechnology and Interdisciplinary Studies, Rensselaer Polytechnic Institute, Troy, New York, United States of America; University of Colorado Denver School of Medicine, UNITED STATES

## Abstract

To better understand some aspects of bone matrix glycation, we used an *in vitro* glycation approach. Within two weeks, our glycation procedures led to the formation of advanced glycation end products (AGEs) at the levels that corresponded to approx. 25–30 years of the natural *in vivo* glycation. Cortical and cancellous bones from human tibias were glycated *in vitro* using either glucose (glucosylation) or ribose (ribosylation). Both glucosylation and ribosylation led to the formation of higher levels of AGEs and pentosidine (PEN) in cancellous than cortical bone dissected from all tested donors (young, middle-age and elderly men and women). More efficient glycation of bone matrix proteins in cancellous bone most likely depended on the higher porosity of this tissue, which facilitated better accessibility of the sugars to the matrix proteins. Notably, glycation of cortical bone from older donors led to much higher AGEs levels as compared to young donors. Such efficient *in vitro* glycation of older cortical bone could result from aging-related increase in porosity caused by the loss of mineral content. In addition, more pronounced glycation *in vivo* would be driven by elevated oxidation processes. Interestingly, the levels of PEN formation differed pronouncedly between glucosylation and ribosylation. Ribosylation generated very high levels of PEN (approx. 6- vs. 2.5-fold higher PEN level than in glucosylated samples). Kinetic studies of AGEs and PEN formation in human cortical and cancellous bone matrix confirmed higher accumulation of fluorescent crosslinks for ribosylation. Our results suggest that *in vitro* glycation of bone using glucose leads to the formation of lower levels of AGEs including PEN, whereas ribosylation appears to support a pathway toward PEN formation. Our studies may help to understand differences in the progression of bone pathologies related to protein glycation by different sugars, and raise awareness for excessive sugar supplementation in food and drinks.

## Introduction

The importance of the advanced glycation end-products (AGEs) formation in biological systems was recognized for the first time in late 1960’s when it was discovered that non-enzymatic processes leading to AGEs formation in human body are similar to the Maillard reaction occurring during food browning at elevated temperatures [1, 2]. It was established then that diabetic patients displayed increased formation of glycosylated hemoglobins [3]. Later, it was determined that AGEs are formed at a slow but constant rate in a healthy human body beginning at early embryonic development, and continue to accumulate with time. *In vivo* accumulation of AGEs has not only been associated with major pathogenic processes in diabetes [4, 5], but also with other health disorders such as atherosclerosis, neurodegenerative diseases [6] and normal aging. For example, pyrraline was detected in brain tissue from patients with Alzheimer disease [7]. Products originating from α-ketoaldehyde transformations such as glyoxal-lysine dimer and methylglyoxal-lysine dimer were identified as major Maillard reaction cross-link products in lens proteins. The concentrations of these two products were significantly elevated in lens proteins of elderly patients [8]. It was also shown that accumulation of AGEs deteriorates mechanical properties and fracture resistance of bone [9–12].

**Fig 1:**
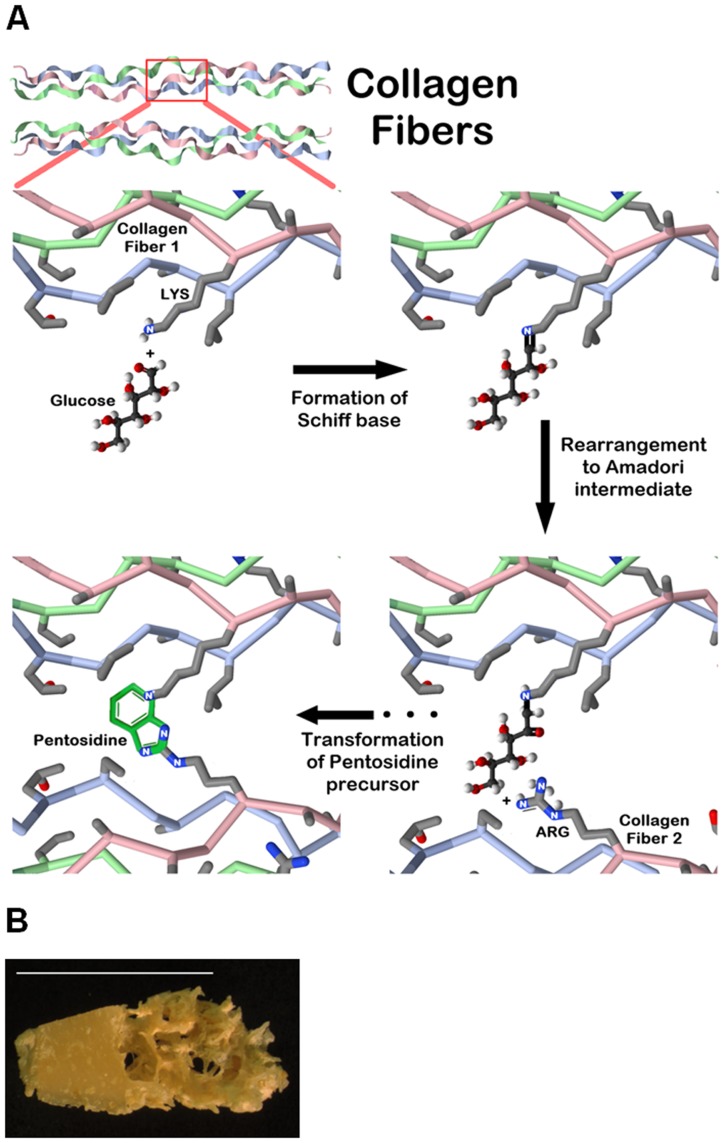
Schematic representation of the main steps of Maillard reaction used to glycate human bone samples. **A**. In the initial step, a given sugar attaches to a free amino group present on the protein surface, and then, through a sequence of different reactions, an advanced glycation end product is formed. As the example, we show pentosidine as the final glycation product. **B**. Glycation process was conducted using spatially matched cortical (left side) and cancellous (right side) bone samples. A bone piece from the 61 year-old donor is shown as the example. The bar corresponds to 1 cm.

The Maillard reaction is remarkably complicated [2]. Based on the *in vitro* studies, the reaction process is traditionally divided into three main steps (Fig. 1*A*). The initial step of the non-enzymatic glycation is the condensation of reducing sugars (in the open chain form) with the unprotonated N-terminal amino acid residues or epsilon amino groups of proteins, lipids, and nucleic acids. As a result, a Schiff base, a reversible and unstable N-substituted glycosylamine, is produced. In this initial step, glucose shows the slowest glycation rate when compared to other reducing sugars. In the next step, the Schiff base undergoes isomerization termed an Amadori rearrangement and converts into an array of more stable Amadori adducts known as ketosamines. Ketosamines undergo further dehydration either to form reductones and dehydro-reductones, or change to short-chain, hydrolytic fission products such as diacetyl, acetol or pyruvaldehyde.

The chemical nature of many AGEs is currently unknown. AGEs form a large group (estimated approx. 750 different AGEs) of complex and very heterogenous compounds which include naturally fluorescent crosslinks such as pentosidine [13, 14], non-fluorescent crosslinking products such as glucosepane [15], glyoxal-lysine dimer (GOLD) and methylglyoxal-lysine dimer (MOLD) [8], or non-fluorescent, non-crosslinking adducts such as carboxymethyl-lysine (CML) [16] and pyrraline [17]. Formation of chemically stable AGEs can permanently alter protein structure and function. Long-lived tissue proteins such as bone matrix collagen accumulate AGEs with age, and hence, contribute to the development of fragile bones [9–12, 18].

Sometimes certain AGEs attract more attention in one research field than the other. For example, carboxymethyl-lysine (CML) was the first AGE identified in food products and therefore is commonly used as a marker of dietary AGEs. In the bone tissue studies, pentosidine (PEN), a mature, naturally fluorescent AGE is typically used as a marker of non-enzymatic glycation of bone matrix proteins. Measurement of PEN can predict vertebral fractures *in vitro* independently of bone mineral density [19]. It was also shown that PEN accounts for 9% of the variance in trabecular ductility [10] and up to 23% of the variation in bone fracture toughness [20]. Several molecularly defined AGEs that are known to accumulate in soft tissues have not yet been studied in bone because of technical constrains imposed by bone mineral on the isolation, identification and quantitation of specific AGEs. In particular, bone mineral solubilizes well at elevated temperatures in strong acids such as hydrochloric acid. AGEs which are resistant to strong acids and high temperature are relatively simple to quantify in bone. Therefore, we selected PEN in order to study potential differences between the glucose- and ribose-based glycation of human cortical and cancellous bone from donors of different age and sex. In addition to sucrose, fructose, lactose and maltose, these two sugars are the most common components of human diet.

Based on the USA economical disappearance data, the average intake of added sugars from all sources was approx. 218 g/day per person in 2000 [21]. The largest single source of sugars in human diet is added sugars consumed in deserts, candies, and most importantly, in soft drinks and other sweetened beverages, all of which are produced using high-fructose corn syrup that typically contains 42, 55 or 90% fructose [22]. Compelling evidence shows that diets high in sugars, in particular fructose and sucrose, can lead to obesity, insulin resistance/glucose intolerance, and dyslipidemia in animals [23–26] and humans [21, 27–35].

Ribose is a naturally occurring monosaccharide essential to every living cell. This sugar is likely the second most abundant carbohydrate in human blood. It is present at approx. 100 μM concentration in human fasting serum which is approx. 50-fold lower than blood glucose concentration in a healthy person [36, 37]. Ribose metabolites contribute to the formation of many important biomolecules such as nucleic acids (RNA and DNA) [38], vitamins (riboflavin) [39] and the key energy storing compound, adenosine triphosphate (ATP) [40]. Most studies focus on the intracellular synthesis of ribose-5-phosphate from glucose [41]. However, cells can also retrieve ribose from extracellular environment for the needs of cellular metabolism [42]. With few exceptions, cells and tissues cannot survive with ribose as their sole carbohydrate source [43–45]. Therefore, it was proposed that ribose may have alternative and distinct roles different from those of glucose in the body.

Ribose metabolite ATP is the most important energy compound in human body. For example, it fuels the process of muscle contraction. After an intensive physical exercise when the ATP pool is depleted, human body restores its ATP levels by converting glucose to ribose, and then to ATP [41]. This process can be speeded up by ingestion of ribose supplements. It has been shown that taking ribose supplements could benefit athletic performance and reduce muscle soreness and stiffness associated with intensive exercise. Therefore, D-ribose is used as the ingredient of sports nutrition products. Currently, ribose is an ingredient in approximately 100 products such as, for example, energy bars (Detour, FastFuel, Marathon) and beverages (SoBe Adrenaline Rush, Vitamin Water, Snapple Antioxidant Water). Moreover, ribose is added in larger quantities to medical food to assist patients with compromised heart function, chronic fatigue syndrome (CFS) and fibromyalgia (FMS) [46]. However, the studies on the influence of ribose-rich supplements on the overall human health, specifically in the context of glycation are very limited.

The role of ribose in *in vivo* and *in vitro* glycation processes has recently attracted a lot of attention after abnormally high levels of D-ribose were detected in the urine of type 2 diabetic patients [47] suggesting that these patients not only suffer from disorders in glucose metabolism but also from ribose metabolism disorders. A few earlier *in vitro* studies established that ribosylation led to protein aggregation [48], significant alteration of the collagen structure [49] as well as the reduced proliferation, increased necrosis and apoptosis of cultured pancreatic islet beta-cells exposed to the ribosylated fetal calf serum [50]. Recent *in vitro* neurotoxicity studies involving ribosylated bovine serum albumin (BSA) showed that misfolded, globule-like aggregates of BSA were highly cytotoxic to neural cells [51]. Extension of these *in vitro* to the *in vivo* studies demonstrated that ribosylation of brain proteins impaired mouse spatial recognition [52]. The aforementioned data clearly support the need for studies on the relationship between ribosylation and the development of different diseases.

Glucose is the key sugar of energy metabolism in living organisms. D-glucose is present in millimolar range in human plasma (on average up to 5 mM in healthy people and 20–50 mM in the plasma of diabetic patients) [16, 36]. Still, glycation of mineralized bone tissues *in vitro* using this sugar has thus far been unsuccessful. This is why ribose is commonly used for *in vitro* glycation of different biological materials including bone [9]. We reasoned that due to the difference in the reactivity between glucose and ribose [53], it may be possible to capture some quantitative and qualitative differences in the formation of certain AGE(s) in bone matrix (i.e., AGE(s) that are naturally fluorescent as well as heat and high-acidity resistant) between the two sugars. This not only could bring new insights into the chemistry of Maillard reaction in bone, but may even influence the amount of sugars added to different food formulations and/or certain energy supplements and drinks [26–28].

Bone differs from all other tissues in a body by being composed largely of a mineral (70–90%) and a small amount of total organic material (10–30%) that contains a uniquely large proportion of collagen (approx. 90%). Non-fibrillar organic matrix comprises a total of approx. 10%, and again within this group of proteins, osteocalcin and osteopontin are present in a large proportion (1 to 2% in a healthy bone). Together with collagen, these major non-collagenous proteins of bone matrix form a scaffold for hydroxyapatite deposition [54]. Osteocalcin and osteopontin have recently begun to be recognized as critical determinants of bone quality and its ability to resist fracture [54–56]. Taken together, collagen and non-collagenous proteins (NCPs) are important contributors to bone quality [56] as both these groups of proteins are the subjects of biochemical modifications (e.g., undesired glycation [57]).

Currently there is no information available on the kinetics of advanced glycation end products and pentosidine formation during *in vitro* glycation of mineralized animal or human bone. It is also unknown to what degree other bone matrix proteins than collagen can undergo glycation. Thus, the objectives of the present study were to glycate *in vitro* human cortical and cancellous bone tissues from donors of different age and sex using either glucose or ribose and to examine the rate and quantity of fluorescent AGEs formation in the extracellular bone matrix. To test the hypothesis of a potential glycation of the major non-collagenous bone matrix proteins, we used osteocalcin as the representative of this protein group. For the first time our study provides the evidence for the formation of significant amounts of pentosidine (PEN) during ribose-based glycation of bone matrix proteins. Interestingly, our *in vitro* glycation studies show that pentosidine is one of the intermolecular crosslinks formed between osteocalcin and its proximal matrix proteins (a manuscript in preparation). We also demonstrated that cancellous bone is more efficiently glycated by both sugars when compared to cortical bone. This may have important implications for current understanding of the progression of bone pathologies related to protein glycation by different sugars such as diabetes, osteoporosis and other sugar-related diseases.

## Materials and Methods

### Chemicals and reagents

If not otherwise stated all chemicals were ultrapure or molecular biology grade. All reagents used for chromatographic separations were HPLC grade. Acetonitrile and acetic acid were purchased from Fisher Scientific (Morris Plains, NJ, USA). Heptafluorobutyric acid was purchased from Sigma-Aldrich (St. Louis, MO, USA). Hydroxyproline Reagent kit was purchased from Bio-Rad (München, Germany). The pentosidine standard was purchased through International Maillard Reaction Society, www.imars.org). Human osteocalcin was purchased from Sigma-Aldrich (St. Louis, MO, USA).

### Human bone samples

To determine natural levels of fAGEs and PEN, tibias (posterior area) from total of 18 human female donors (young 35.0 ± 15.0, middle age 60.0 ± 10 and elderly donors 80.0 ± 15.0 years old) served as the source of cortical bone tissue samples. For the *in vitro* glycation experiments, tibias (posterior area) from human female (23, 59 and 86 years old) and male (25, 61 and 87 years old) donors served as the source of cortical and cancellous bone (Fig. 1*B*). The specimens obtained from the centralized National Disease Research Interchange (NDRI) biobank were known to be free of osteoarthritis, diabetes and other metabolic bone diseases. They were also certified to be free of HIV and hepatitis B. Collected bone pieces were repeatedly washed in cold distilled water until the washings were free of contaminating blood and other impurities that are not the part of bone matrix [30]. After freeze-drying, the specimens were stored at -80°C until their use.

### *In vitro* glycation process using ribose (ribosylation)

Each bone sample (5 or 10 mg) was placed into a vial containing sterile Hank’s buffer pH 6.8–7.0 (Sigma, St. Louis, MO, USA) supplemented with 0.6 M ribose, 1.25 mM ε-amino-n-caproic acid, 5 mM benzamidine, 10 mM N-ethylmaleimide, 30 mM HEPES, and 0.5 M CaCl_2_. The 0.6 M concentration of ribose is the standard concentration of this sugar used for glycation of bone samples *in vitro* [9, 18]. Ribose was omitted from the buffer that served as a glycation solution for control samples. All samples were incubated at 37°C. The pH of the incubated solutions was monitored daily and maintained between 6.8 and 7.2 using 0.1 M hydrochloric acid or 0.1 M sodium hydroxide to lower or raise the pH, respectively. Next, the samples were dialyzed extensively against water for 48 to 72 hours to remove free sugar. After dialysis, the samples were lyophilized over-night (ON) and stored at -80°C until their analysis. Osteocalcin was ribosylated according to the same protocol as the bone samples.

### *In vitro* glycation process using glucose (glucosylation)

The glycation of bone samples with glucose was performed according to a similar procedure for that of ribose. However, before the transfer of each bone sample (5 or 10 mg) into the sterile Hank’s buffer, the samples were lyophilized with glucose and incubated under vacuum for 2 days. Also, the Hank’s buffer was supplemented with 0.6 M glucose (sterilized using filtration) instead of ribose. All other components of the buffer were the same as for ribosylation. The samples that served as controls did not have glucose added into the Hank’s buffer. All samples were incubated at 37°C under controlled pH ranging between 6.8 and 7.2, and then, dialyzed extensively against water as described above. In the final step, the samples were lyophilized ON and stored at -80°C until their use.

### Kinetics of the initial phase of fluorescent AGEs and pentosidine formation

Glucosylation and ribosylation were performed as described above. However, for each kinetics experiment, bone pieces (1.0–1.1 mg) of the same bone part (Fig. 1*B*) from a given donor were used. Every day for 7 days, and then, from the 10^th^ day every 3 or 4 days, one bone piece was taken out from the Hank’s buffer for future analysis. The respective sampling was conducted under aseptic conditions, i.e., inside a laminar flow hood. The bone pieces were stored at -80°C until their use. For the remaining bone pieces, the glycation continued as described above.

### Measurement of fluorescent AGEs

Direct acid hydrolysis of the glycated bone samples, glycated osteocalcin (OC) and non-glycated controls was performed in 6N HCl (100 μl/mg bone) at 110°C for 20 hrs. After completion of the hydrolysis, the hydrolysates were centrifuged and the supernatants were divided into portions. Each portion was transferred into a clean tube and used directly for the assays or stored at -80°C as needed. Since the defined amounts of OC were taken out for glycation from the stock solution (c_stock_ = 0.1 μg OC/μl), only the measurement of each hydrolysate fluorescence was needed in order to calculate the levels of fluorescent AGEs (fAGEs) per mmol of OC.

The assay to measure fAGEs in bone matrix has two parts. The first one is the fluorometric assay for determination of fAGEs content “in-bulk.” This assay is based on the measurement of natural fluorescence of AGEs as compared to the fluorescence of the quinine (Q) standards (the stock solution: 10 mg/mL quinine per 0.1 N sulfuric acid) at 360/460 nm excitation/emission using a microtiter-plate (MT-plate) reader (model Infinite 200; Tecan). The second assay component is the colorimetric assay for determination of collagen content in bone samples through the measurement of hydroxyproline concentration.

Hydroxyproline was used to prepare the standard curve for the colorimetric assay. All solutions were made fresh directly before their use. The assay was initiated by addition of chloramine-T solution to hydroxyproline standards (the stock solution: 2 mg/mL L-hydroxyproline per 0.001 N HCl) and to the hydrosylates of bone samples. These solutions were then incubated at room temperature (RT) for 20 minutes. Subsequently, 3.15 M perchloric acid solution was added to the samples and 5 min incubation at RT followed. Next, the p-dimethylaminobenzaldehyde solution was added and the samples were incubated for 20 minutes at 60°C. Finally, all the standards and the samples were cooled down to RT in darkness. The absorbance was measured at 570 nm using the MT-plate reader (model Infinite 200; Tecan). Collagen content was calculated based on the determined amount of hydroxyproline [58, 59]. The amounts of fluorescent AGEs were expressed in the terms of unit of fluorescent quinine per unit of collagen (e.g., fAGEs [μmol Q/mmol Col]).

### Measurement of pentosidine by UPLC

PEN was measured using ultra-high performance liquid chromatography [58, 59]. Two analyses were performed on each bone hydrolysate, one to measure pentosidine content, and a second to determine hydroxyproline content that was further used to calculate collagen concentration. Since the defined amounts of OC were used for glycations, only the measurement of PEN content was needed.

**Fig 2:**
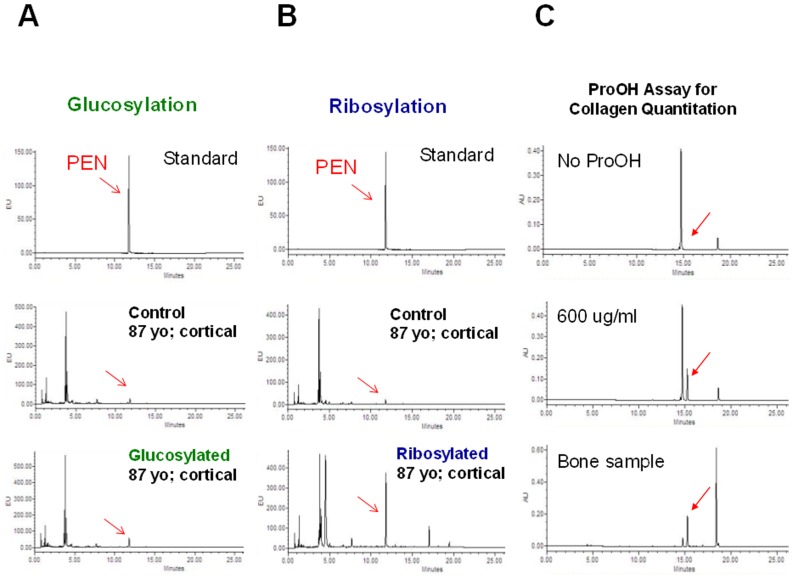
Examples of UPLC chromatograms. **A**. Identification of PEN (shown by red arrow) in the glucosylated human cortical bone samples. **B**. Identification of PEN (shown by red arrow) in the ribosylated human cortical bone samples. **C**. The UPLC chromatogram with the peak of ProOH (shown by red arrow) used for determination of collagen contents. Similar amounts of the samples were injected to the column. Notably, the chromatogram obtained from the analysis of the ribosylated sample contains several peaks that are not present in the glucosylated sample.

Before the UPLC analysis, each hydrolysate was dissolved in 1% n-heptafluorobutyric acid (HFBA). PEN was separated using an Acquity UPLC machine (Waters Corp., Milford, MA, USA) equipped with the reverse-phase Acquity UPLC HSS T3 column (1.8 μm; 2.1 x 100 mm). The column flow rate and temperature were 0.400 ml/min and 40°C, respectively. Solvent A contained 0.06% HBFA in 18 ohms pure water, and solvent B was composed of 50: 50 (v: v) mixture of solvent A: acetonitrile. Prior the use, the column was equilibrated using 10% solvent B. Gradient of 10 to 50% of solvent B (from 8 to 20 min) was used for the separation of PEN. The elution of PEN was monitored for fluorescence emission at 385 nm after excitation at 335 nm (Fig. 2*A* and *B*). PEN was quantified using a standard curve.

### Measurement of hydroxyproline by UPLC

Hydroxyproline content was determined using reagents from the HPLC assay kit (Bio-Rad Labratories GmbH, Müchen, Germany), but the mobile phase solvents and conditions were developed specifically for the UPLC separation. The column flow rate and temperature were 0.400 ml/min and 60°C, respectively. The 0 to 50% gradient of acetonitrile was achieved by mixing 100% acetonitrile (solvent B) with a buffer composed of 0.3% acetic acid and 0.6% triethylamine, pH 4.50 (solvent A). The elution of the derivatized hydroxyproline was monitored at 471 nm (Fig. 2*C*). The amount of hydroxyproline was determined using a standard curve. The amount of collagen was calculated assuming 300 nmol of hydroxyproline in 1 mol of collagen (e.g., PEN [μmol PEN/mmol Col]) [58, 59].

## Results

**Fig 3:**
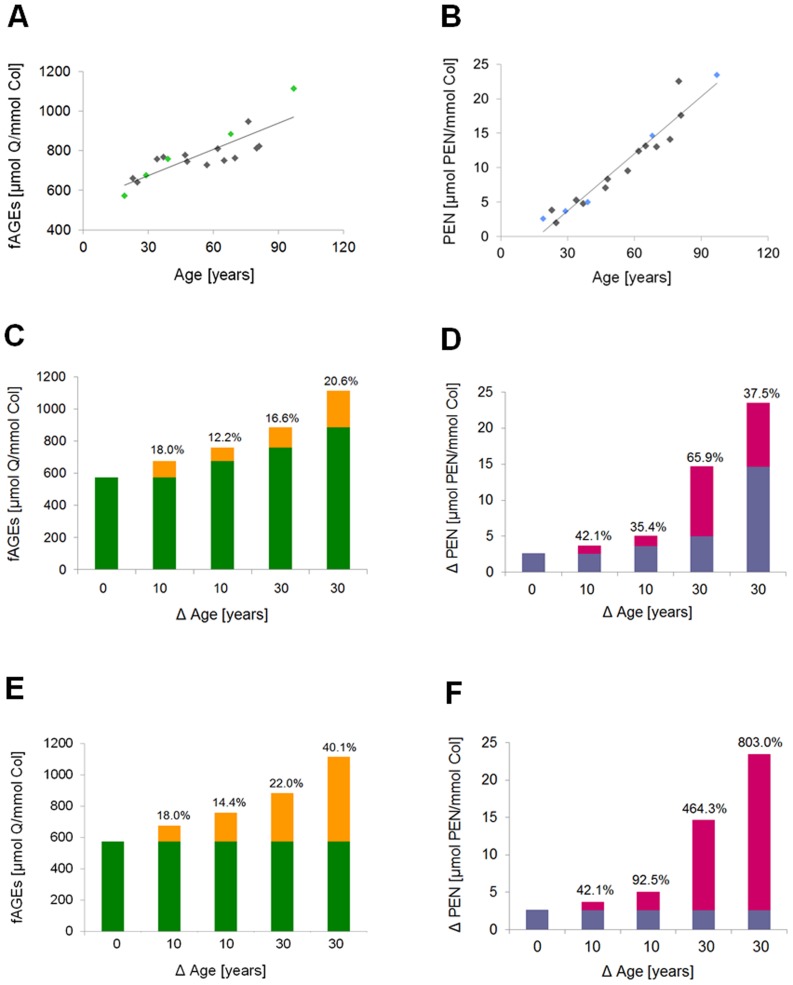
Natural levels of fAGEs and PEN in human cortical bone matrix. **A**. The levels of bone-matrix fluorescent AGEs increase with the increasing age of human donors. **B**. Bone-matrix levels of PEN increase with the increasing age of human donors. **C**. The percentage of fAGEs increase during 10 years (for 19–29 and 29–39 years old donors) and 30 years (for 39–68 and 68–97 years old donors). **D**. The percentage of PEN increase during 10 years (for 19–29 and 29–39 years old donors) and 30 years (for 39–68 and 68–97 years old donors). **E**. The percentage of fAGEs increase between the youngest available donor and other older donors (i.e., 19–29 [10 years], 19–39 [20 years], 19–68 [±50 years], 19–97 [±80 years]). **F**. The percentage of PEN increase between the youngest available donor and other older donors (i.e., 19–29 [10 years], 19–39 [20 years], 19–68 [±50 years], 19–97 [±80 years]).

In order to study the rate and quantity of fluorescent AGEs formation in bone matrix, we selected bone pieces that contained similar initial levels of fAGEs and PEN (Table 1). Natural levels of AGEs and PEN in bone show some variation not only between healthy donors of the same age [18], but also within a given healthy donor. Such differences are normal and can be explained, for example, by various life styles and/or genetic traits. We were most interested in in the levels of fluorescent AGEs formed *in vitro* between the 7^th^ and 10^th^ day, because our earlier work showed that the levels of fAGEs and PEN formed during this period of time corresponded to approx. 25 to 30 years of natural *in vivo* glycation of bone tissue (Fig. 3). We confirmed that the accumulation of fAGEs (Fig. 3*A*) and pentosidine (Fig. 3*B*), both normalized to the collagen contents in cortical bone from human tibia, increased with the donors’ age. In order to determine the extent of the fAGEs and PEN increase over selected decades of human life, we chose 19, 29, 39 68 and 97 years old donors (discerned in green in Fig. 3*A* and in blue in Fig. 3*B*). We calculated to what degree (expressed as the percentage) fAGEs and PEN increased between the selected decades (i.e., 19–29 [10 years], 29–39 [10 years], 39–68 [±30 years], 68–97 [±30 years]; Fig. 3*C* and Fig. 3*D*, respectively) as well as between the age of the youngest available donor (i.e., 19 year old) and the age of other older donors (i.e., 19–29 [10 years], 19–39 [20 years], 19–68 [±50 years], 19–97 [±80 years]; Fig. 3*E* and Fig. 3*F*, respectively).

**Table 1:** The determined natural, *in vivo* levels of fluorescent AGEs and pentosidine in cortical and cancellous bone tissue originating from healthy young, middle-age, and elderly donors.

Donors Age [years]	fAGEs		PEN	
	[mmol Quinine/mmol Collagen]		[μmol PEN/mmol Collagen]	
	Cortical	Cancellous	Cortical	Cancellous
20–25	1.7–2.1	2.0–2.4	2.1–3.0	2.9–3.0
60–65	1.8–2.3	2.1–2.4	9.0–10.0	5.1–8.0
85–90	1.9–2.5	2.0–2.9	9.6–13.2	10.2–13.8

Using the aforementioned information, for example, we produced the *in vitro* levels of fAGEs and PEN in bone samples originating from middle-age donors that would match the physiological levels of AGEs observed either in elderly people (85 years and older) or middle-age diabetic patients (60–64 years old). Since we investigate different characteristics of aging bone, we regularly use the time-frame of 7^th^ and 10^th^ days and the developed *in vitro* glycation conditions (as described in Materials and Methods) in order to mimic aging process and/or conditions of diabetes in our studies on the role of glycation in the resistance of bone to fracture.

### Difference between glucose- vs. ribose-based formation of fluorescent AGEs in bone matrix

**Fig 4:**
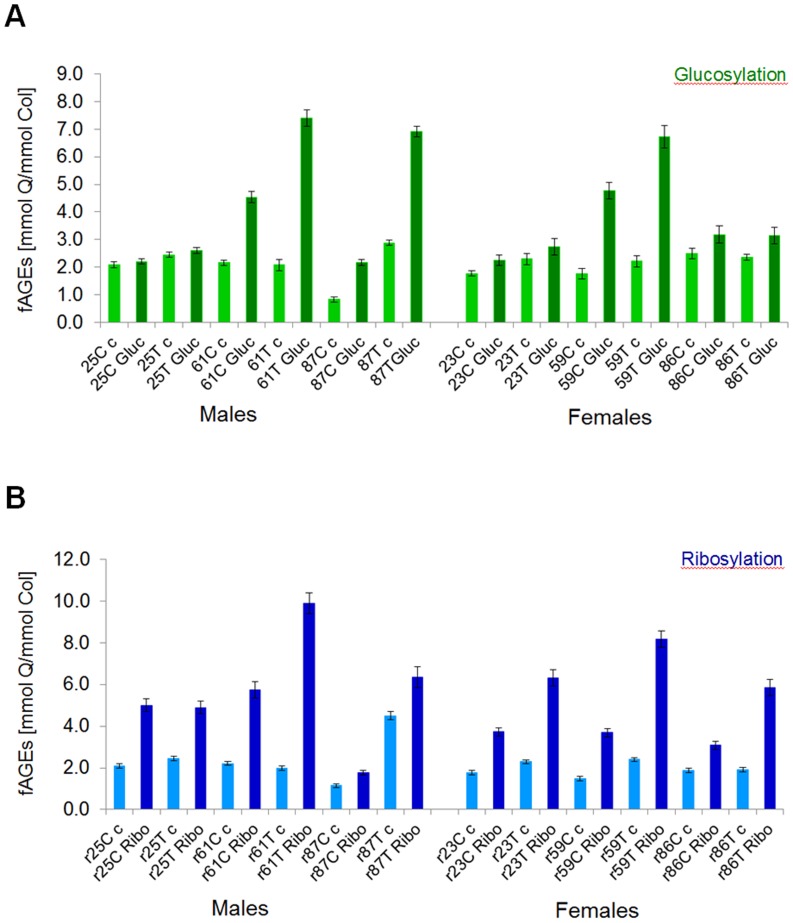
Comparison of the glucosylation (A) and ribosylation (B) through the determined fluorescent AGEs content. Fluorescent AGEs were quantified in glycated (for 7 days) human cortical (C) and cancellous (T) bone samples originating from young, middle-age and elderly male and female donors. The abbreviation code for the samples is as follows: 25 C c corresponds to **25** years old **c**ortical bone of **c**ontrol and 25 C Gluc corresponds to **25** years old **c**ortical bone of **g**lucose. Ribosylated samples have **r** in front of the age, and Gluc is replaced by **Ribo** for ribose (r25 C c and r25 C Ribo).

We established that typically glycation with ribose produced approx. 2-fold higher levels of fAGEs as compared to glycation using glucose (on average 6.0 mmol quinine (Q)/ mmol collagen (Col) for ribosylation vs. average 3.3 mmol Q/mmol collagen for glucosylation) (Fig. 4). Ribosylation led also to approx. 2- to 2.5-fold increase in the fAGEs content between glycated and non-glycated (controls) bones in all types of tested human bone tissues that were glycated for the same length of time (Fig. 4*B*). Age of donors influenced the outcome of *in vitro* glycation, in particular in young donors. Thus, ribosylation led to the formation of higher levels of fAGEs than glucosylation in young male and female donors (Fig. 4). Conversely, the levels of fAGEs in bone tissues of middle-age and elderly donors were high after either glucosylation or ribosylation (Fig. 4). Considering the donor’s sex, the levels of fAGEs were typically slightly higher in male than female bone tissues on the 7^th^ day of glycation.

### Formation of fluorescent AGEs differs between human cortical and cancellous bone

The amount of fluorescent AGEs formed during the first 7 days of glucosylation and ribosylation was significantly higher in cancellous than cortical bone (Fig. 4).

*In vitro* glucosylation led to the formation of higher levels of fAGEs in bone tissues of middle-age (on average for the 61 year-old male 4.5 and 7.4 mmol Q/mmol collagen for glucosylated cortical and cancellous bone, respectively; on average for the 59 year-old female 4.6 and 6.8 mmol Q/mmol collagen for glucosylated cortical and cancellous bone, respectively) and elderly donors, but not in young donors (on average for young male 2.2 and 2.6 mmol Q/mmol collagen for glucosylated cortical and cancellous bone, respectively; on average for young female 2.3 and 2.7 mmol Q/mmol collagen for glucosylated cortical and cancellous bone, respectively). Notably, the bone tissues of young donors showed the lowest propensity for glycation using either glucose (see above) or ribose (on average for the young 25 year-old male 4.9–5.0 and 4.7–5.1 mmol Q/mmol collagen for ribosylated cortical and cancellous bone, respectively; on average for the young 23 year-old female 3.7 and 6.3 mmol Q/mmol collagen for ribosylated cortical and cancellous bone, respectively) (Fig. 4*A* and *B*) when compared to the data collected for all older donors.

### Pentosidine as the key fluorescent AGE formed in bone matrix by ribosylation

**Fig 5:**
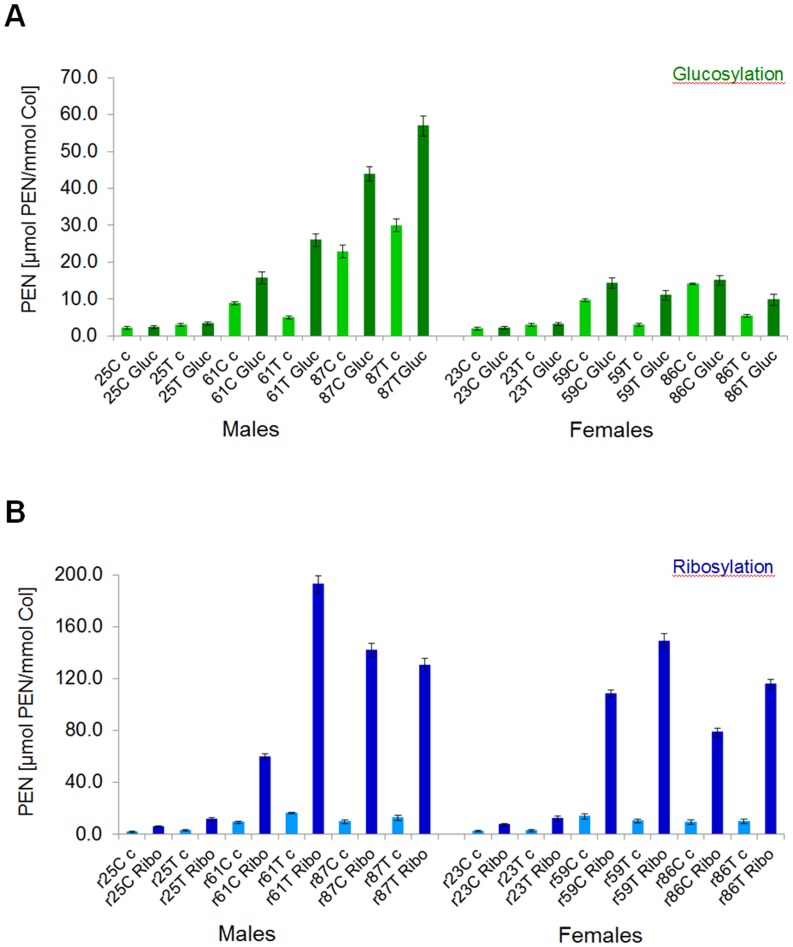
Quantification of pentosidine in the glucosylated (A) and ribosylated (B) bone samples. Fluorescent PEN was quantified in glycated (for 7 days) human cortical (C) and cancellous (T) bone samples originating from young, middle-age and elderly male and female donors. The abbreviation code for the samples is the same as described in the legend for Fig. 3.

The levels of PEN were measured using highly sensitive UPLC methods (Fig. 2). We established that the amount of PEN formed within the first 7 days of glycation was approx. 3- (young donors) to 6-fold (elderly donors) higher for ribose than glucose (Fig. 5). Considering approx. 2-fold difference in fAGEs formation between the two sugars, this indicates that the amount of PEN constituted a significant portion of fAGEs formed during ribosylation. Moreover, like for fAGEs, the levels of PEN in bone samples originating from the older donors were significantly higher (up to approx. 193 μmol PEN/mmol collagen for ribosylation vs. up to approx. 57 μmol PEN/mmol collagen for glucosylation) than in the young donors (Fig. 5).

Another interesting observation was that while the levels of PEN formed during glucosylation were quite similar between young males and females, they differed more pronouncedly between older men and women (Fig. 5*A*). As opposed to glucosylation, ribosylation led to significant increase of PEN levels in bone samples from all age groups of donors (Fig. 5*B*).

### Higher levels of pentosidine formation in human cancellous than cortical bone

Both glucosylation and ribosylation led to a more pronounced increase of PEN levels in cancellous than cortical bone of all donors (Fig. 5).

Pentosidine levels were typically approx. 1.5- to 1.8-fold higher after glucosylation of bone samples originating from the young male and female donors as compared to the control samples. The highest levels of PEN were formed in the middle-age and elderly male and female donor samples (2.0-fold for glucosylated cortical and 3.0-fold for glucosylated cancellous bone).

Ribosylation led to approx. 2.5 to 3.0-fold higher level of PEN in the young donors (approx. 6.2–9.0 μmol PEN/mmol collagen) and 5- to 6-fold higher level of PEN (approx. 109–193 μmol PEN/mmol collagen) in the older donors as compared to the spatially matched controls.

### Kinetics of the initial phase of fluorescent AGEs formation in bone matrix

**Fig 6:**
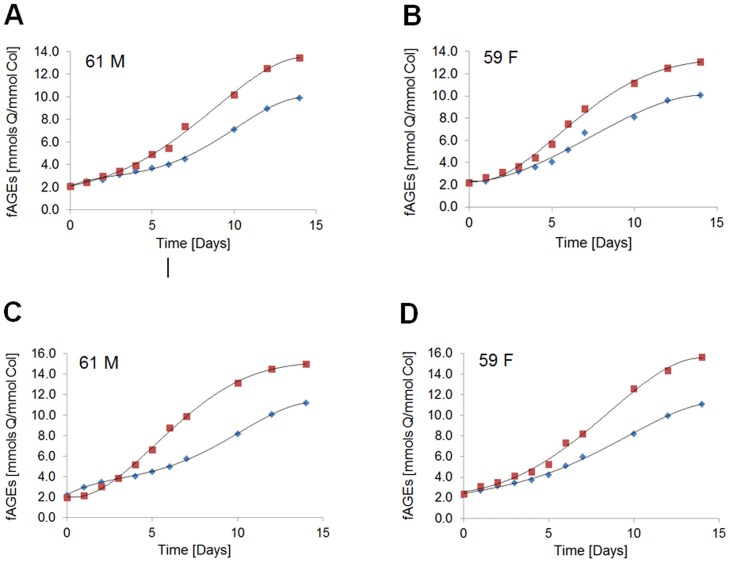
Kinetics of fluorescent AGEs formation. The content of fAGEs was determined in human cortical (blue diamonds) and cancellous (red squares) bone after glycation using glucose (**A** and **B**) or ribose (**C** and **D**) for the male (61 M) and the female (59 F) donor.

Kinetics of the fAGEs formation was followed for cortical and cancellous bone tissues originating from the 61 year-old male and the 59 year-old female donor. The levels of fAGEs were measured for glucosylation (Fig. 6*A* and *B*) and ribosylation (Fig. 6*C* and *D*). We observed that after approx. one day of the induction period (a lag phase), the formation of fluorescent AGEs increased steadily in cortical as well as cancellous bone until beginning to approach the plateau around the 14^th^ day of incubation (Fig. 6). The half-time of fluorescent AGEs formation in the bone matrix was 6 to 7 days for ribose and 20 to 22 days for glucose.

Like for the end-point measurements (i.e., on the 7^th^ day; Fig. 4), glucosylation and ribosylation led to more pronounced formation of fAGEs in cancellous than cortical bone of male and female donors. Interestingly, some differences referring the levels of fAGEs that were observed between the sexes at the early stages of glycation (Fig. 4) diminished when formation of fAGEs had begun to approach the plateau (Fig. 6*A* and *C* vs. *B* and *D*).

### Kinetics of the initial phase of pentosidine formation in bone matrix

The levels of PEN were measured in the same acidic hydrolysates that were used for the determination of fAGEs concentration. After *in vitro* glycation, a pronounced difference in the levels of PEN was observed between cancellous and cortical bone.

**Fig 7:**
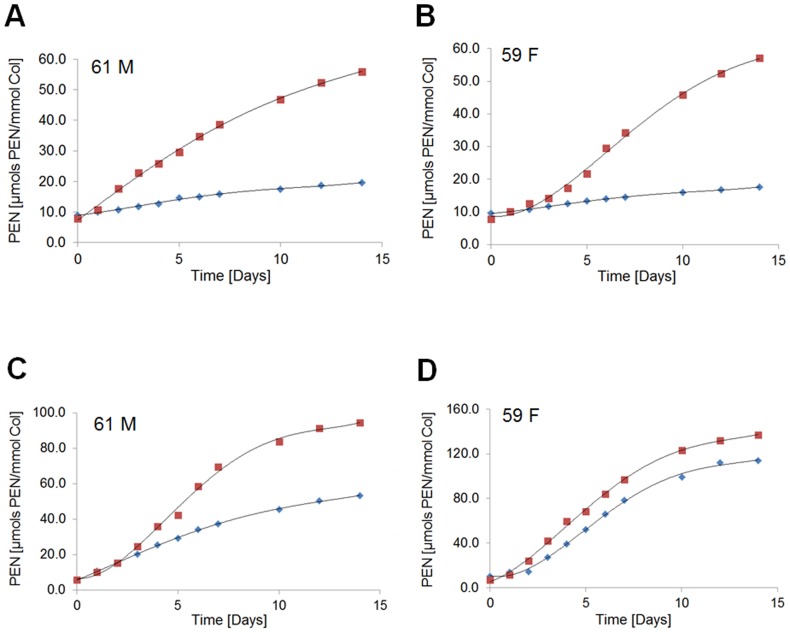
Kinetics of pentosidine formation. The content of PEN was determined in human cortical (blue diamonds) and cancellous (red squares) bone after glycation using glucose (**A** and **B**) or ribose (**C** and **D**) for the male (61 M) and the female (59 F) donor.

We established that the formation of PEN displayed a steady and significant increase in the cancellous bone samples originating from both the male and female donor (average 4- to 5-fold increase for glucose and 12- to 14-fold increase for ribose) (Fig. 7). The half-times of PEN formation using ribose or glucose were similar to those determined for fAGEs (i.e., approx. 6 to 7 days for ribose and approx. 17 to 19 days for glucose).

The amount of PEN formed in cortical bone during kinetic experiments differed significantly between glucose and ribose (average 1.5- to 1.7-fold increase for glucose vs. 6.5- to 7-fold increase for ribose) (Fig. 7). Interestingly, the levels of PEN formed during *in vitro* ribosylation were lower in cortical bone from the male (Fig. 7*C*) than the female donor (Fig. 7*D*). Typically the cortical bone samples from the male donor showed approx. 2-fold lower levels after ribosylation (Fig. 7*C*). Conversely, the cancellous bone showed slightly higher level of PEN in the female (Fig. 7*D*) than the male (Fig. 7*C*) donor.

### Glycated osteocalcin

We determined that like collagen, OC undergoes glycation which leads to the production of fluorescent AGEs. The analysis of the acidic hydrolysates of OC using UPLC revealed the presence of pentosidine. We established that pentosidine is one of the intermolecular crosslinks formed between osteocalcin and its proximal matrix proteins (a manuscript in preparation).

## Discussion

The fundamental importance of glycation in bone health has begun to be recognized recently due to its significant role in the deterioration of mechanical properties and fracture resistance of bone [9–12]. In order to better understand the impact of the Maillard reaction on bone aging and diabetes related fragility fractures, as well as to develop effective therapeutic methods to prevent accumulation of AGEs in different tissues, it is essential to understand the contribution of different sugars to the formation of AGEs. Investigation of changes in mechanical properties of bone tissue due to glycation relies considerably on the *in vitro* methods. However, the methods that were initially developed for *in vitro* glycation of purified proteins require approx. 3 to 9 months of incubation [16]. Mimicking physiological conditions [16], for example with the respect to the glucose concentration in the *in vitro* glycation reactions, helped to determine natural progression of AGEs formation for single proteins (e.g., BSA, ribonuclease A, lysozyme) and established that crosslinking and insolubility are the major changes that happen to proteins that undergo glycation. However, the use of the aforementioned methods for *in vitro* glycation of mineralized bone matrix would be impractical for several reasons, in particular, the input of time spent on the experiments. Therefore, we focused our studies on two reaction parameters: the sugar concentration (was increased to c = 0.6 M) and the length of incubation time, both of which effectively control the levels of AGEs produced in bone matrix. Using our approach we could, for example, convert younger bone (e.g., from a 40 year old donor) characterized by lower levels of AGEs into a bone mimicking the bone of a donor of the specific, older age (e.g., 65 year old or older human). Our experimental strategy permitted investigation of certain aspects of bone matrix glycation, which otherwise could not be studied. Thus, for the first time we report that in addition to some similarities, there is a pronounced difference in the quantity and likely quality of the fluorescent AGEs formation, including PEN, between glucose-based and ribose-based *in vitro* glycation of mineralized bone matrix.

We demonstrated that ribose is a potent glycation agent as compared to glucose, because it showed ability to glycate bone tissues from young donors. Formation of significant amounts of AGEs during bone matrix ribosylation can be explained, for example, by facile reaction of more reactive pentose than hexose with free functional groups of amino acids and the subsequent conversion into various AGEs precursors into mature AGEs. Moreover, we observed a significant quantitative difference in PEN formation between glucose (approx. 2.0% and 4.4% of PEN in fAGEs formed in cortical and cancellous bone, respectively) and ribose (approx. 4.8% and 8.4% of PEN in fAGEs formed in cortical and cancellous bone, respectively) as compared to the corresponding difference between fAGEs measured “in-bulk.” We propose that ribosylation may support a pathway towards PEN formation. We also infer that there may be a difference in the capacity of the two processes to form some AGEs. Thus, in the case of some AGEs, there may be a quantitative difference in their formation, in which PEN can serve as the example. Other AGEs may be formed only during glucosylation or ribosylation. Taken together, these intriguing observations add to the complexity of the AGEs formation driven by the Maillard reaction and require more studies.

Another interesting observation was that albeit glucosylation and ribosylation were conducted using mineralized bone, the produced levels of fAGEs and PEN were high. Both glucosylation and ribosylation led to a higher formation of fluorescent AGEs in cancellous than cortical bone, in particular in the middle-age and elderly male and female donors. One of the reasons for the higher formation of AGEs appears to be the structural differences between cortical (Fig. 1*B* left) and cancellous (Fig. 1*B* right) bone. It is likely that compact cortical bone serves as a better barrier against glycating sugars than naturally porous cancellous bone. This conclusion is also supported by our observation that *in vitro* glycated cortical bone samples from the middle-age and elderly donors had higher levels of fAGEs than those from the young donors. Among other issues, the amount of mineral phase decreases with aging and this significantly increases porosity of bone tissues [60]. As the protective role of mineral begins to decline, glycation of bone matrix proteins becomes more pronounced.

The kinetic studies of fAGEs and PEN accumulation confirmed that cancellous bone is more prone to the formation and accumulation of the glycation products. These studies also revealed surprisingly fast conversion of sugars into AGEs when the conditions favor the Maillard reaction. The differences in the levels of fAGEs and PEN produced during glycation of bone tissues originating from male donors as compared to female donors may be explained, for example, by the prior *in vivo* glycation history (“local age’) of the dissected bone samples.

Pentosidine is considered to be the well-defined AGE of sugar origin. In the first step of PEN formation, the aldehyde group of the open-chain glucose attaches to the free amino groups of such amino acids as lysine or arginine. In the last step of PEN formation, the transformation of pentosidine precursor(s) into mature PEN involves oxidation [14]. Since oxidation processes facilitate the conversion of pentosidine precursor, pentosinane, into mature PEN [14] and these processes increase in bone with aging, they could enhance the *in vivo* formation of PEN, and potentially other AGEs, which are the products of carbohydrate oxidation. These AGE products are known as “advanced glycoxidation end products” (AGOEs) and represent a subgroup of AGEs [14]. This could also explain the observed significant increase in the *in vivo* formation of fAGEs and PEN in donors of 65 or older (Fig. 3).

Bone matrix collagen comprises 90% of organic matrix and its amino acid sequence is rich in lysines and arginines, the key amino acids involved in the formation of PEN [14, 15]. Thus, the sequence space of collagen available for glycation is very large due in part to its relatively simple fiber structure that helps to expose amino acids onto the protein surface. In addition, bone matrix collagen interacts with a number of different non-collagenous proteins (NCPs), for example, osteocalcin and osteopontin [54, 56]. Lysines, arginines, valines and tyrosines are the most commonly glycated amino acids [17]. For example, *in vivo* glycation is the major cause of heterogeneity in human hemoglobin [61]. In red cells, glucose reacts predominantly with the N-terminal valine of the β-chains, to a lesser extent with the N-terminal valine of the α-chains, and with several Ɛ-amino lysines [61]. Interestingly, among amino acids that compose human OC, there are several arginines, valines and tyrosines that could potentially undergo glycation. It has already been established that the N-terminal tyrosine of human OC can become glycated *in vivo* [57]. Thus, OC in bone could become post-translationally glycated to a variable extent depending upon the age and the local glucose concentration. Our study revealed that pentosidine is one of the intermolecular crosslinks formed between osteocalcin and its proximal matrix proteins (a manuscript in preparation). We propose that as a result of close structural proximity of the key amino acids in collagen with respect to osteocalcin [56, 62, 63], there are multiple opportunities for the formation of various AGEs between these two proteins as well as other NCPs in bone matrix. The detection of OC with the PEN crosslink supports the aforementioned conclusions.

The discussed results also turn attention to some practical aspects of our studies, in particular, the influence of excessive sugar addition to energy drinks and food on general health. Commonly, ribose is used to improve athletic performance and the ability to exercise by boosting muscle energy. Studies to evaluate the effectiveness of ribose in improving athletic performance as well as the role of *in vivo* ribosylation on the overall health are needed.

In conclusion, our studies have given new insights into glycation processes occurring in human cortical and cancellous bone samples when two different sugars were used. As different sugars compose a substantial part of human diet, our studies may help to understand certain bone and other organs pathologies that are related to protein glycation.
